# Bone regeneration is associated with the concentration of tumour necrosis factor-α induced by sericin released from a silk mat

**DOI:** 10.1038/s41598-017-15687-w

**Published:** 2017-11-14

**Authors:** You-Young Jo, HaeYong Kweon, Dae-Won Kim, Kyunghwa Baek, Min-Keun Kim, Seong-Gon Kim, Weon-Sik Chae, Je-Yong Choi, Horatiu Rotaru

**Affiliations:** 1 Sericultural and Apicultural Division, National Institute of Agricultural Science, RDA, Wanju, 55365 Republic of Korea; 2 Department of Oral Biochemistry, College of Dentistry, Gangneung-Wonju National University, Gangneung, 28644 Republic of Korea; 3 Department of Oral Pharmacology, College of Dentistry, Gangneung-Wonju National University, Gangneung, 28644 Republic of Korea; 4 Department of Oral and Maxillofacial Surgery, College of Dentistry, Gangneung-Wonju National University, Gangneung, 28644 Republic of Korea; 5 Analysis Research Division, Daegu Center, Korea Basic Science Institute, Daegu, 41566 Republic of Korea; 6 School of Biochemistry and Cell Biology, BK21 Plus KNU Biomedical Convergence Program, Skeletal Diseases Analysis Center, Korea Mouse Phenotyping Center (KMPC), Kyungpook National University, Daegu, 41944 Korea; 7 Department of Cranio-Maxillofacial Surgery, “Iuliu Hatieganu” University of Medicine and Pharmacy, Cluj-Napoca, 400001 Romania

## Abstract

To understand the osteogenic effect of the middle layer of the silk cocoon, sericin was examined for its cellular effects associated with tumor necrosis factor-α (TNF-α) signaling in this study. The fragmented sericin proteins in the silk mat were evaluated for the TNF-α expression level in murine macrophages. The concentration of protein released from silk mats was higher in the outermost and the innermost layers than in the middle layers, and the protein released from the silk mat was identified as sericin. The level of TNF-α in murine macrophages was dependent on the applied concentration of sericin, and the expression of genes associated with osteogenesis in osteoblast-like cells was dependent on the applied concentration of TNF-α. In animal experiments, silk mats from the middle layers led to a higher regenerated bone volume than silk mats from the innermost layer or the outermost layer. If TNF-α protein was incorporated into the silk mats from the middle layers, bone regeneration was suppressed compared with unloaded silk mats from the middle layers. Accordingly, silk mats from the silk cocoon can be considered to be a fragmented sericin-secreting carrier, and the level of sericin secretion is associated with TNF-α induction and bone regeneration.

## Introduction

Tooth loss is caused by periodontal disease, trauma, and tumours. Alveolar bone is the supporting structure of the tooth and, accordingly, loss of a tooth is followed by the loss of alveolar bone^1^. The regeneration of alveolar bone is important for oral rehabilitation, such as in dental implant installation^2^, and many types of biomaterials have been used for the regeneration of alveolar bone. Among many options, guided bone regeneration (GBR) is a simple technique that uses a membrane^3^. The original concept of the GBR membrane was as a barrier that inhibited the infiltration of epithelial cells into bony defects. The recently developed membrane used for GBR is not a passive barrier but acts in active osteogenesis as a drug carrier^4^. A collagen membrane with bone morphogenic protein is actively under investigation for the maximum gain of new bone formation^4^.

Silk fibroin is produced by *Bombyx mori* and is studied for use in scaffolding in bone grafts^5^. Because silk fibroin is a bio-inert material and has an osteogenic property, silk fibroin has been considered for use in GBR membranes^6^. The electrospinning technique or precipitation method can be used during the production of silk fibroin as a GBR membrane^6,7^. Although the electrospun technique can be used in mass production, the use of toxic solvents and the need for a large facility present disadvantages^7^. Compared with the electrospun technique, the precipitation method is simple. However, the film-type membrane produced by the precipitation technique is fragile in dry state and easily solubilized in normal saline^6^.

Recently, a new eco-friendly method was introduced in which the natural cocoon of the silkworm was used as a GBR membrane^8^. The silkworm cocoon is composed of multiple layers that can be easily separated by peeling^9^. Membranes originating from the middle layer of the cocoon show a similar level of bone regeneration to those of commercially available collagen membranes^8^. Several comparative reports have been published on natural cocoon-derived GBR membranes^10,11^. These studies showed that a thicker membrane obtained from the middle layer of the cocoon shows better bone regeneration than a thinner one^10^. Comparing the unprocessed cocoon with the membrane-originated middle layer, the middle layer group shows better bone regeneration^11^. Although all membranes from the silk cocoon have the same origin, the different behaviors of the GBR membrane in bone regeneration have not been explained. The differences between the silkworm cocoon layers lie in the content of sericin protein^12^.

The content of sericin 1 in the cocoon increases from the inner layer to the outer layer^12,13^. The content of sericin 3 in the cocoon decreases from the inner layer and then increases to the outer layer^13^. Accordingly, removals of the outermost and the innermost layers mean the removal of the sericin 1/3-rich area. If any type of sericin is beneficial for bone regeneration, a degumming process should not be required for bone tissue engineering. However, the unprocessed silk cocoon shows poor bone regeneration compared with the middle layer of the silk cocoon^11^. Considering that sericin is the bonding protein among fibroin fibres, excess sericin should be easily solubilized in water. Therefore, the concentration of protein released from the silk mat is higher in the innermost layer and the outermost layer of the silk cocoon than in the middle layer^14^. Released sericin from the silk cocoon might influence cells adjacent to the graft. To understand the osteogenic effect of the middle layer silk mat, sericin was examined for its cellular effects associated with tumor necrosis factor-α (TNF-α) signaling in this study.

TNF-α is a key cytokine involved in acute inflammation^15^. The level of TNF-α is increased at the implantation site of a foreign graft in response to an immune reaction^15^. Blood-originated cells, including macrophages, are the main source of TNF-α^16^. Bone formation around a foreign graft is associated with the level of TNF-α^17^. A high level of TNF-α is a strong activator of osteoclasts and is involved in bone resorption^16,17^. Controversies have emerged related to the role of a low level of TNF-α in new bone formation^17,18^. Silk fibroin is known to induce a low level of TNF-α at the graft site^19^. However, the relationship between sericin and TNF-α induction is still controversial. Because the degumming process and origin of sericin differ in each study, the direct comparisons between studies are difficult. If sericin released from the silk mat induced a different level of TNF-α leading to activation or suppression of new bone formation, the mechanism explaining the different behavior of each layer of silk cocoon in bone regeneration could be revealed.

In this study, the silk cocoon was separated into 4 layers with the same thickness, and the concentration of sericin released from the surface of each silk mat was measured. A sericin solution from each silk mat was applied to macrophages, and the level of TNF-α was measured. In addition to released sericin, sericin from degumming products was also applied to macrophages, and the level of TNF-α was measured and compared with the results from each silk mat. The effect of TNF-α on osteoblasts was also tested. Finally, each silk mat was applied to calvarial defects in an animal model, and new bone formation was analysed. Accordingly, the purpose of this study was to analyse the effect of TNF-α induced by each silk mat layer on bone regeneration.

## Results

### Strength of each silk mat and the concentration of released protein

The tensile strength of layer 1 was the highest among the 4 silk mat groups (Table 1). The difference in the tensile strength among the groups was statistically significant (P = 0.014). In the post hoc test, the tensile strength was significantly higher in the layer 1 group compared with the layer 3 group (P = 0.020). No statistically significant differences were noted between the layer 2 and the layer 3 groups (P > 0.05). In the case of tensile strain, that of layer 4 was the highest among the 4 silk mat groups (Table 1). The difference in the tensile strain among the groups was statistically significant (P = 0.001). In the post hoc test, the tensile strain was significantly higher in the layer 4 group compared with the layer 2 group and the layer 3 group (P = 0.002 and 0.014, respectively). No statistically significant differences were observed between the layer 2 and the layer 3 groups (P > 0.05).

**Table 1:** Physical property of each silk mat.

	Tensile strength (MPa)	*P-value	Tensile strain (%)	**P-value
Layer 1	48.11 ± 14.89	—	18.10 ± 3.77	NS
Layer 2	28.64 ± 10.23	NS	9.81 ± 1.85	0.002
Layer 3	24.73 ± 8.87	0.020	12.40 ± 4.97	0.014
Layer 4	28.33 ± 7.66	NS	21.72 ± 4.86	—

(*P-value for comparison with Layer 1, **P-value for comparison with Layer 4, NS: not significant).

**Figure 1:**
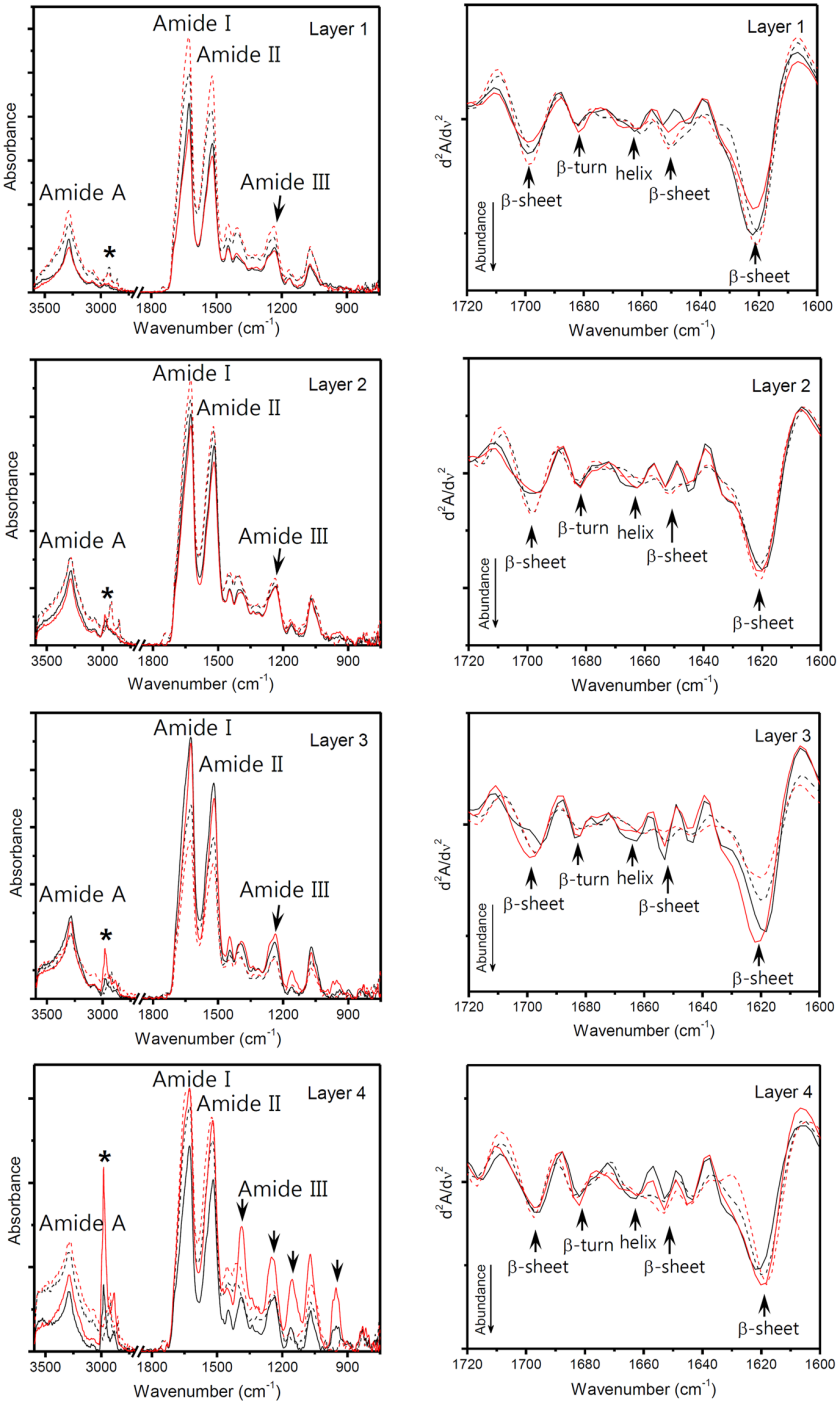
Results of ATR-FT-IR spectra. ATR-FT-IR spectra of the silk mat from different layers before (dotted line) and after (solid line) sonication. Second-derivative spectra of the amide I peak before (dotted line) and after (solid line) sonication. The inside (black line) and outside (red line) of the silk mat were also measured. Characteristic vibrational absorptions of silk protein are shown at 1626 (amide I), 1520 (amide II) and 1236 cm^−1^ (amide III). The composition of the secondary protein structure does not show significant change regardless of layer numbers and the inside and outside of the layers. Sonication treatment satisfactorily retained the composition. Additionally, the C–H stretching peaks become strengthened after sonication in the outside layers in the order of layer 1 to layer 4 (indicated by an asterisk). For layer 4 of the silk, the C–H stretching peak was maximized, and several additional peaks were observed at 1387, 1248, 1153 and 953 cm^−1^ after sonication treatment (indicated by arrows). These enhanced IR absorption peaks are considered related to released protein after sonication.

Figure 1 shows the characteristic vibrational absorptions of the silk protein at 1626 (amide I), 1520 (amide II), and 1236 cm^−1^ (amide III). The distinct absorption at 3280 cm^−1^ can be assigned to hydrogen-bonded N–H stretching (amide A). The absorptions in the region of 2800–3000 cm^−1^ are due to C–H stretching modes. Particularly, it is well known that the amide I peak is highly sensitive to protein secondary structures^20,21^. The second derivatization of the amide I absorption can reasonably resolve the sub-component secondary structures of silk protein. In this study, silk protein consists of a β-sheet (1620, 1650, and 1699 cm^−1^) structure as a major component, as well as β-turn (1682 cm^−1^) and helix (1663 cm^−1^) structures as minor components. The composition of the secondary protein structure does not show significant changes with layer number and location inside or outside of the layers. The sonication treatment retained the secondary protein structure composition. Additionally, one notable point was that the C–H stretching peaks were strengthened after sonication in the outside layers in the order of layer 1 to layer 4 (indicated by an asterisk). For layer 4, the C–H stretching peak was maximized, and several additional peaks were observed at 1387, 1248, 1153, and 953 cm^−1^ after sonication treatment (indicated by arrows). The 1387 cm^−1^ peak is assigned to O–H bending, and the other three absorption peaks are assigned to C–O stretching. These enhanced IR absorption peaks are considered related to the protein released after sonication.

**Figure 2:**
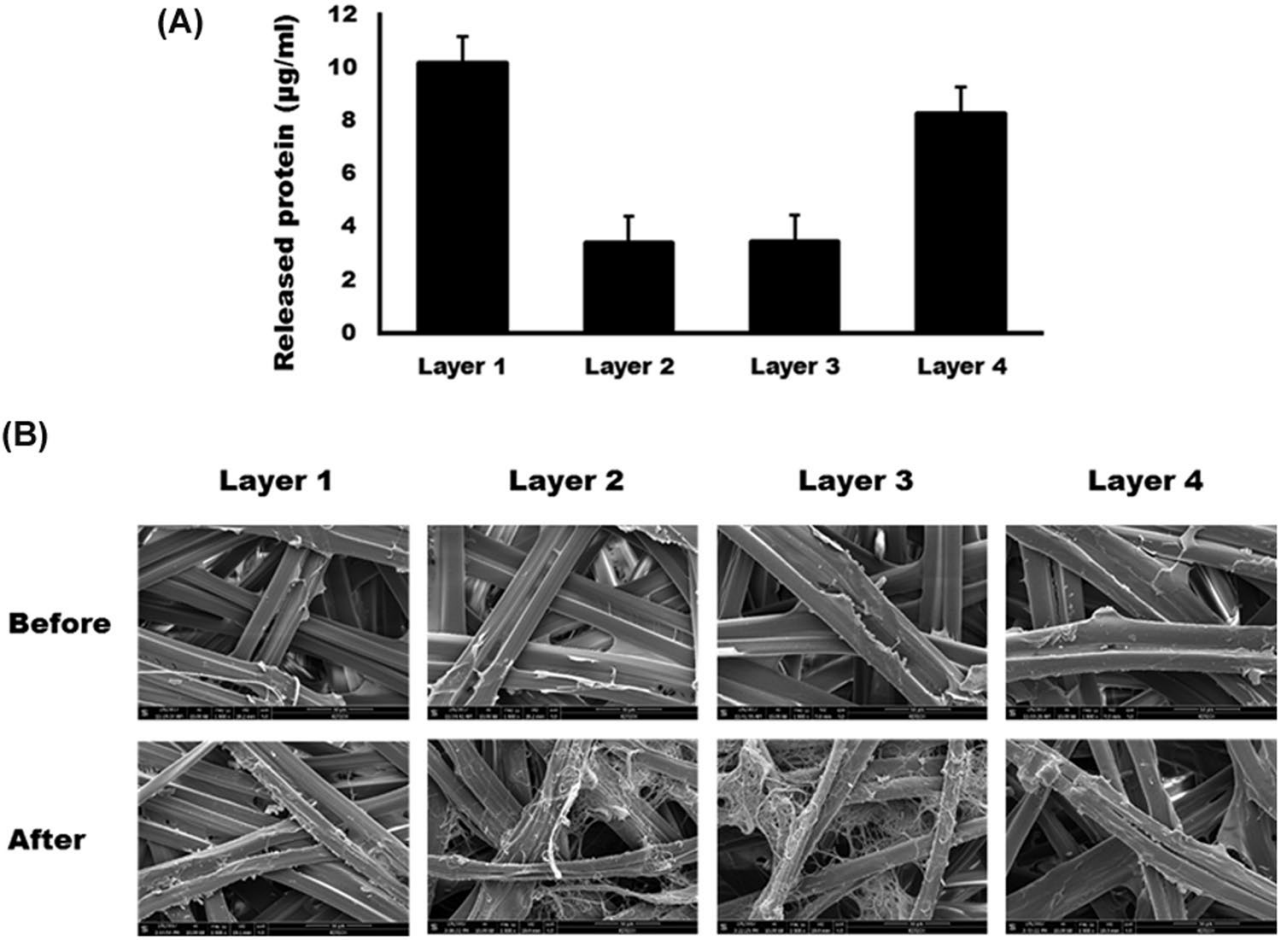
(**A**) Protein concentration in saline after sonication of the silk mat from each layer. The concentrations of protein were 10.16 ± 0.08 μg/ml, 3.38 ± 0.01 μg/ml, 3.44 ± 0.02 μg/ml, and 8.26 ± 0.21 μg/ml for layer 1, layer 2, layer 3, and layer 4, respectively. The concentration of released protein was significantly higher in the layer 1 group and the layer 4 group compared with the layer 2 group and the layer 3 group (P < 0.001 for all comparisons). (**B**) Comparison of SEM images of the silk mat before and after sonication. The dissociated silk fibroin fibre caused by dissolution of the bonding protein was prominent in the layer 2 and the layer 3 groups. Despite the high amount of bonding protein release, the main fibres appeared intact in the layer 1 and the layer 4 groups.

The total protein concentration in saline was highest in the layer 4 group. The protein concentrations were 10.16 ± 0.08 μg/ml, 3.38 ± 0.01 μg/ml, 3.44 ± 0.02 μg/ml, and 8.26 ± 0.21 μg/ml for layer 1, layer 2, layer 3, and layer 4, respectively (Fig. 2A). The difference in the concentration of the released protein among groups was statistically significant (P < 0.001). In the post hoc test, the concentration of released protein was significantly higher in the layer 1 group and the layer 4 group compared with the layer 2 group and the layer 3 group (P < 0.001 for all comparisons). However, no statistically significant differences were found between the layer 2 and the layer 3 groups (P > 0.05). Comparing the SEM images of the silk mat before and after sonication, dissociated silk fibroin fibre caused by the dissolution of bonding protein was prominent in the layer 2 and the layer 3 groups (Fig. 2B). Despite the high amount of bonding protein release, the main fibres appeared to be intact in the layer 1 and the layer 4 groups.

Soluble proteins from the silk mat were identified using two-dimensional gel electrophoresis and LC MS/MS analysis (Supplementary Fig. 1). In two-dimensional electrophoresis, large protein clusters were identified at pH 3, and their molecular weights were between 15 and 50 kDa. Only fragments of sericin were present in the selected area, and the other protein was not identified.

### Sericin increases the expression of TNF-α in RAW264.7 cells

**Figure 3:**
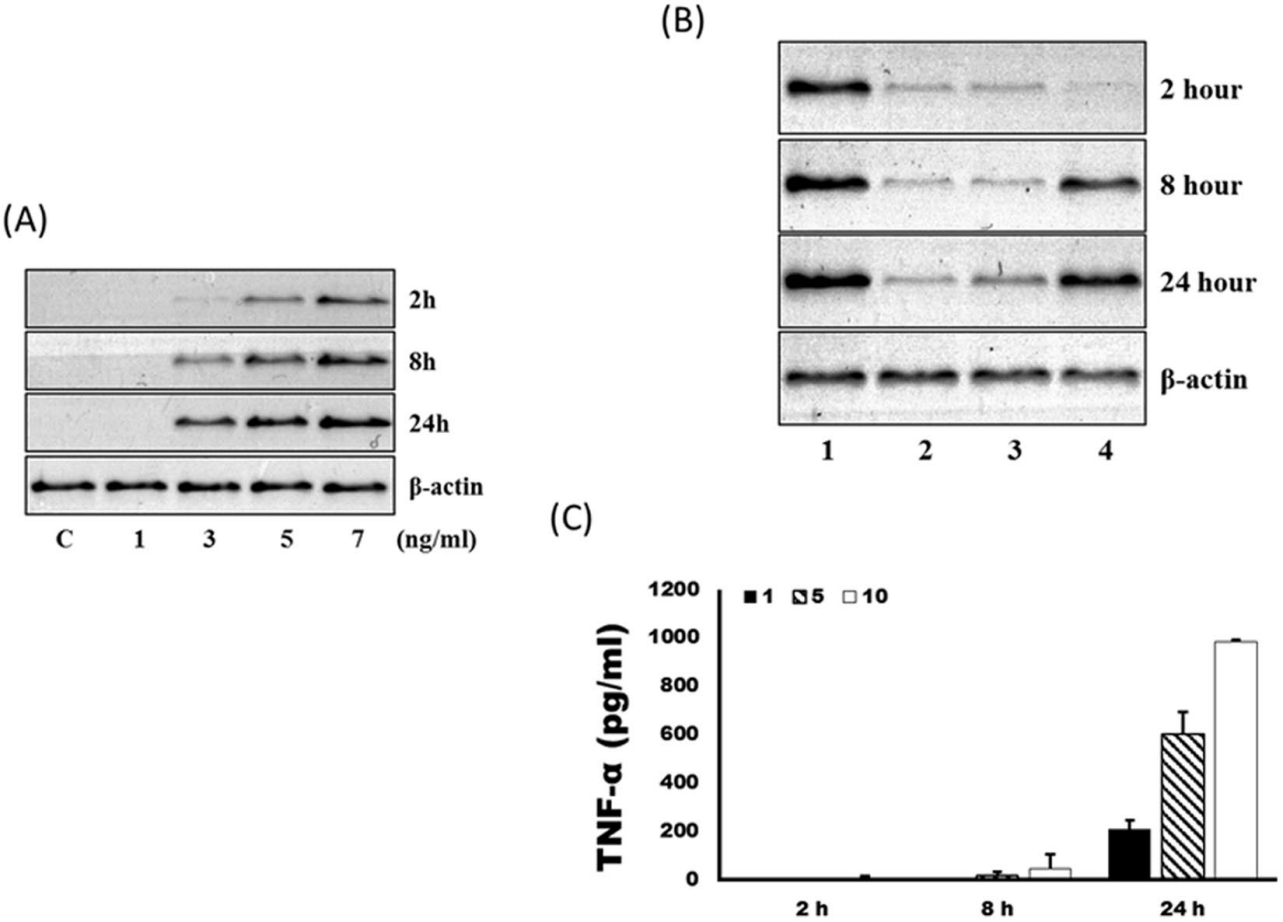
Sericin-induced TNF-α expression in RAW264.7 cells. (**A**) The expression level of TNF-α was increased by the administration of sericin in a dose-dependent manner. (**B**) The protein solution of silk mat from each layer was administered to RAW264.7 cells. Because the concentration of dissolved protein was different for each silk mat, the expression level of TNF-α in each silk mat was also different. The expression level of TNF-α was highest in the layer 1 group followed by that of the layer 4 group. (**C**) According to the ELISA results, TNF-α was not detected in 1 to 5 ng/ml of sericin at 2 h. However, the concentrations of TNF-α at 24 h were 209.07 ± 35.59, 602.97 ± 88.55, and 982.71 ± 8.30 pg/ml for the 1, 5 and 10 ng/ml sericin groups, respectively. The difference among groups was statistically significant (P < 0.001).

When sericin was administered to RAW264.7 cells, the expression level of TNF-α was increased in a dose-dependent manner (Fig. 3A). The concentration of solubilized sericin was different for each silk mat, and, accordingly, the expression level of TNF-α in each silk mat was also different. The expression level of TNF-α was highest in the layer 1 group, followed by the layer 4 group (Fig. 3B). The expression level of TNF-α was low in the layer 2 and the layer 3 groups. According to the ELISA results, TNF-α was not detected in 1 to 5 ng/ml of sericin at 2 h (Fig. 3C). However, the concentrations of TNF-α at 24 h were 209.07 ± 35.59, 602.97 ± 88.55, and 982.71 ± 8.30 pg/ml for the 1, 5, and 10 ng/ml sericin groups, respectively. The difference among groups was statistically significant (P < 0.001).

### Low concentrations of TNF-α enhance osteogenic gene expression in C2C12 cells

**Figure 4:**
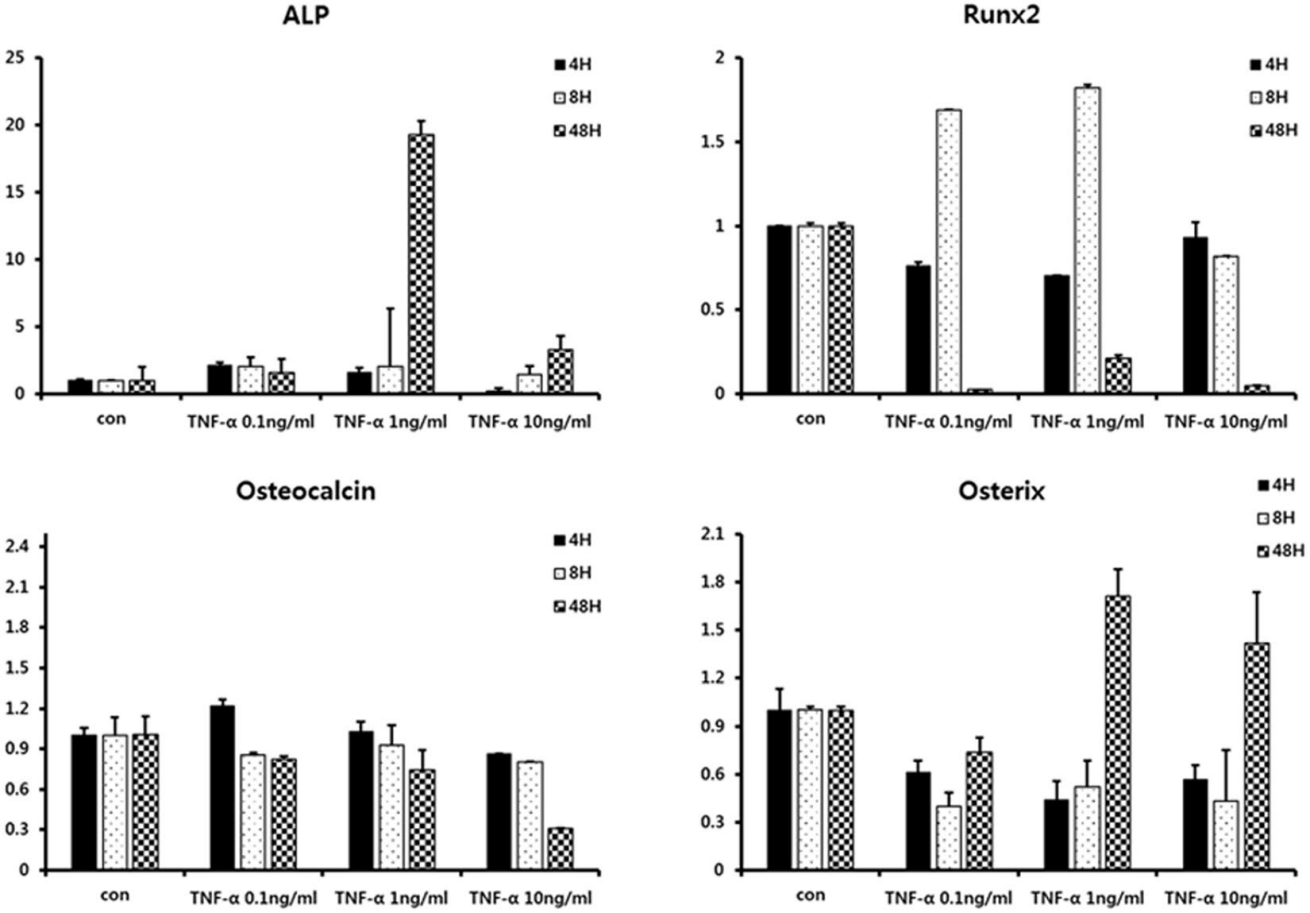
Results of quantitative RT-PCR. To confirm the effect of TNF-α on osteogenic gene expression in C2C12 cells, the cells were incubated for 0, 4, 8 and 48 h in the presence of various concentration of TNF-α (0.1 to 10 ng/ml). At 4 h, an increase in ALP mRNA expression level was observed when the cells were treated with low concentrations of TNF-α (0.1 and 1 ng/ml), in contrast to the results for 10 ng of TNF-α treatment. The levels of Runx2 mRNA expression increased from 8 h when the cells were treated with low concentrations of TNF-α (0.1 and 1 ng/ml). In the case of osteocalcin, the level of mRNA expression was not significantly different among groups. At 48 h, the level of ALP, osterix mRNA expression was increased in 1 ng/ml of TNF-α treatment.

To confirm the effect of TNF-α on osteogenic gene expression in C2C12 cells, cells were incubated for 0, 4, 8, and 48 h in the presence of various concentrations of TNF-α (0.1 to 10 ng/ml). When cells were treated with low concentrations of TNF-α (0.1 and 1 ng/ml), an increase in the ALP mRNA expression level was observed at 4 h in contrast to the results from treatment with 10 ng of TNF-α. The significant increase in ALP mRNA expression with low concentrations of TNF-α (0.1 and 1 ng/ml) was still observed up to 48 h (Fig. 4).

When cells were treated with low concentrations of TNFα (0.1 and 1 ng/ml), the levels of Runx2 mRNA expression increased after 8 h. In the case of Runx2, the effect of a low concentration of TNFα on mRNA expression was not sustained until late time points. In the case of osteocalcin, the level of mRNA expression after treatment with low concentrations of TNF-α (0.1 and 1 ng/ml) remained largely unchanged or slightly decreased compared with that of the untreated control, whereas the level of mRNA expression after treatment with 10 ng/ml TNF-α decreased remarkably over time. Western blot assay showed increases in the protein expression of Runx2 and osteocalcin in C2C12 cells incubated with low concentrations of TNF-α (0.1 and 1 ng/ml) at 48 h (Supplementary Fig. 2).

### The layer 2/3 group showed the highest bone volume and TNF-α immuno-staining similar to the unfilled control

**Figure 5:**
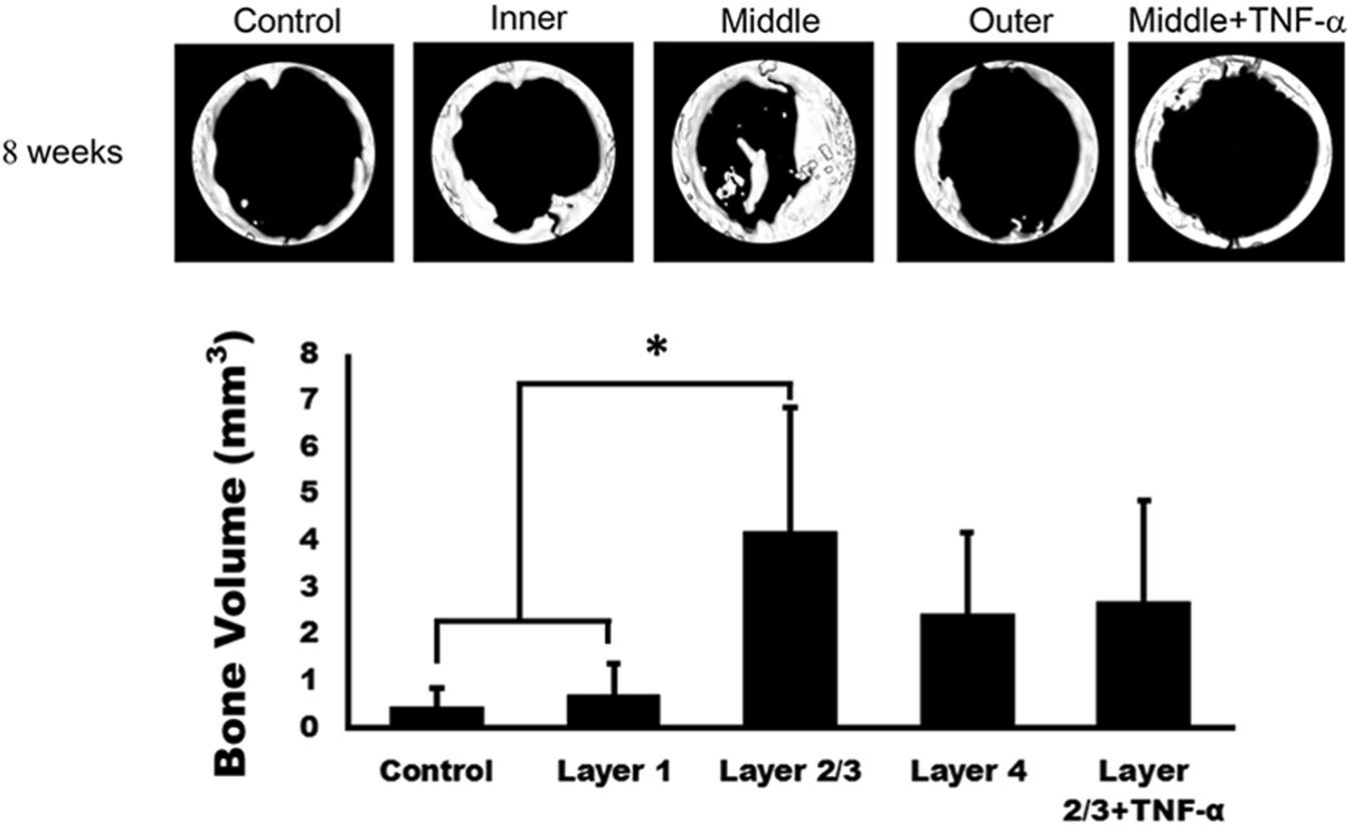
Results of μ-CT analysis. Radiologic analysis revealed that after 8 weeks of implantation, the bone volume in each silk mat group showed a significant difference (P = 0.007). In the post hoc test, the bone volume was significantly increased in the layer 2/3 group compared with the unfilled control group and the layer 1 group (*P = 0.018 and 0.022, respectively). No statistically significant differences were observed between the layer 2/3 + TNF-α group and the other groups (P > 0.05).

The results of μ-CT analysis are shown in Fig. 5. The radiologic analysis revealed that after 8 weeks of implantation, the bone volume in each silk mat group showed a significant difference (Fig. 5; P = 0.007). The bone volumes in the unfilled control, layer 1, layer 2/3, layer 4, and layer 2/3 + TNF-α were 0.44 ± 0.41 mm^3^, 0.68 ± 0.68 mm^3^, 4.18 ± 2.66 mm^3^, 2.42 ± 1.76 mm^3^, and 2.91 ± 2.03 mm^3^, respectively, at 8 weeks after the operation. The unfilled control group and the layer 1 group reflected only partial bone filling from the margins of the defects. In the post hoc test, the bone volume was significantly increased in the layer 2/3 group compared with the unfilled control and the layer 1 groups (P = 0.018 and 0.022, respectively). No statistically significant differences were noted between the layer 2/3 + TNF-α group and the other groups (P > 0.05).

**Figure 6:**
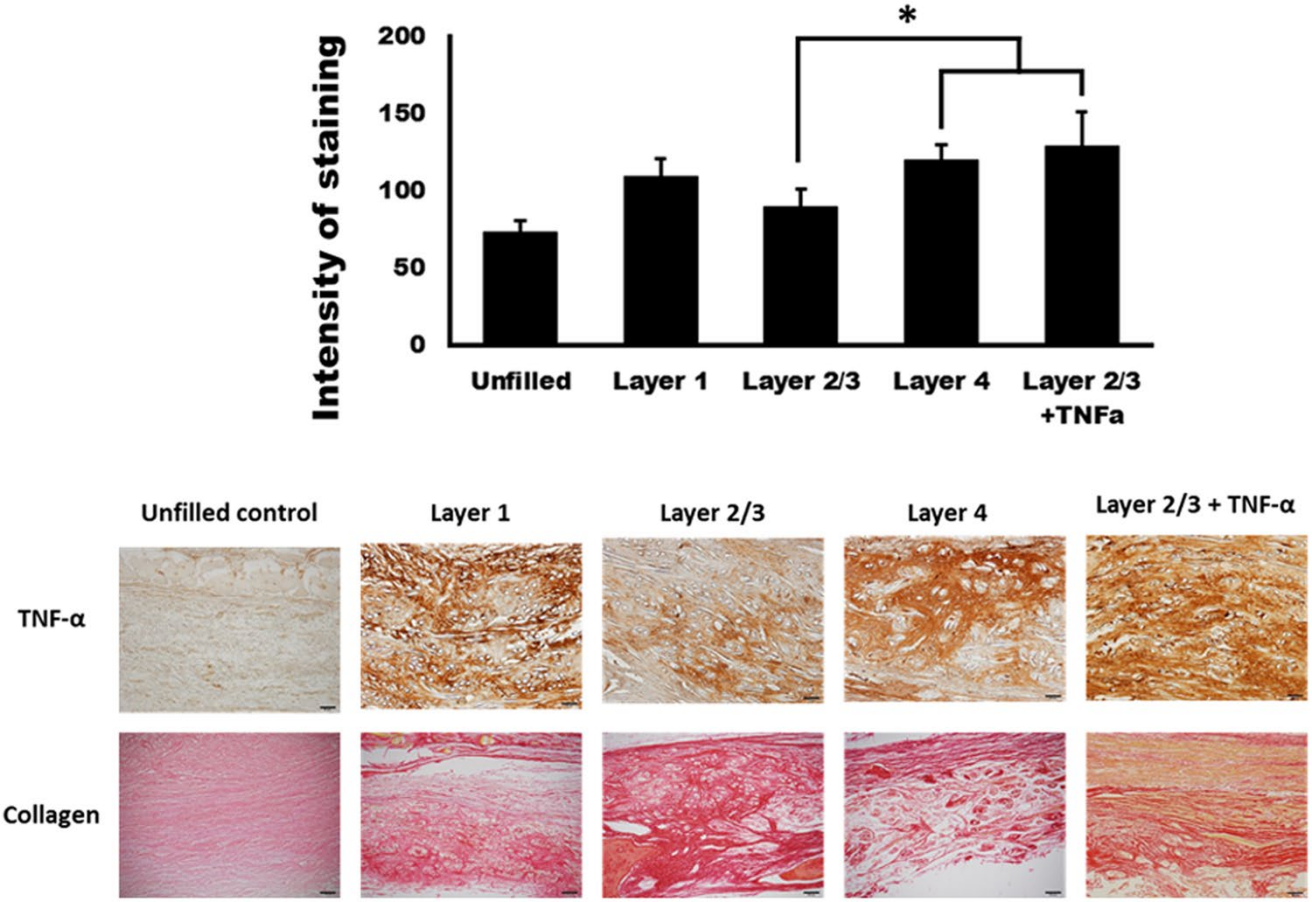
Histological findings. The intensity of TNF-α staining in each silk mat group showed a significant difference (P < 0.001). In the post hoc test, the intensity of TNF-α staining was significantly lower in the layer 2/3 group compared with the layer 4 group and the layer 2/3 + TNF-α group (*P = 0.025 and 0.001, respectively). Sirius staining demonstrated that the collagen-enriched regions were more prominent in the layer 2/3 group.

The intensities of TNF-α staining in the unfilled control, layer 1, layer 2/3, layer 4, and layer 2/3 + TNF-α were 73.11 ± 7.49, 109.48 ± 11.46, 89.70 ± 11.79, 119.86 ± 9.64, and 129.32 ± 21.79, respectively (Fig. 6). The intensity of TNF-α staining in each silk mat group showed a significant difference (P < 0.001). In the post hoc test, the intensity of TNF-α staining was significantly lower in the layer 2/3 group than in the layer 4 and layer 2/3 + TNF-α groups (P = 0.025 and 0.001, respectively). No statistically significant differences were found between the layer 2/3 group and the unfilled control group (P > 0.05). Sirius staining demonstrated that the collagen-enriched regions were more prominent in the layer 2/3 group (Fig. 6). Low inflammation was visualized in the layer 2/3 group, and the expression level of TNF-α was also lower in the layer 2/3 group (Fig. 6).

## Discussion

The silkworm cocoon is composed of multiple layers of silk mat. In this study, the silk cocoon was separated into 4 layers. The concentration of released protein was highest in the layer 1 group, followed by the layer 4 group (Fig. 2). The released protein was mainly identified as sericin (Supplementary Fig. 1), and sericin increased the expression level of TNF-α in RAW264.7 cells in a dose-dependent manner (Fig. 3). Accordingly, the layer 1 group and the layer 4 group showed higher levels of TNF-α in RAW264 cells than the layer 2 and the layer 3 groups (Fig. 3). Application of low levels of TNF-α increased the level of ALP and Runx2 in osteoblast-like cells, but this effect was not observed with high levels of TNF-α (Fig. 4). *In vivo* experiments also demonstrated that the layer 2/3 group showed greater bone regeneration than the unfilled control group and the layer 1 group (Fig. 5). Bone regeneration in the layer 2/3 group was reversed by a high level of TNF-α loading (Fig. 5).

The content of sericin 1 increases from the inner layer to the outer layer of the silkworm cocoon^13,22^. The content of sericin 3 in the cocoon decreases from the inner layer and then increases to the outer layer^13^. Seroin 1 is a small sized (11–13 kDa) anti-microbial protein^23^ and increases from the outer layer to the inner layer of the silkworm cocoon^13^. The content of fibroin is similar among layers, but the relative abundance of proteins is higher in the innermost and in the outermost layer^13^. Therefore, the concentrations of released protein were higher in layer 1 and layer 4 (Fig. 2A). The concentration of sericin is acutely increased at the outer layer of the silkworm cocoon by more than 30%^22^. Although the molecular weight of sericin is greater than 200 kDa, when it is solubilized in water from the silk mat, it is fragmented^24^. Fragmented sericin is amorphous and has a low molecular weight^24^, but because sericin is a bonding protein, it can reunite after fragmentation. For this reason, no specific protein spot was noted from two-dimensional electrophoresis analysis (Supplementary Fig. 1B). The difference in tensile strength among the silk mat groups could be explained by considering the content of sericin and porosity. Sericin has lower tensile strength compared to fibroin^25^. The porosity is highest at the outermost layer and decreases into the inner layer^26^. Accordingly, the tensile strength was higher in layer 1 than in the other layers (Table 1).

Sericin is a degumming product of the silkworm cocoon and is treated as industrial waste^27^. The solubility of sericin is temperature dependent, and sericin is soluble in hot water^28^. In addition to high temperature, acidic solutions, alkali solutions and urea can be used to extract sericin from the silkworm cocoon^29^. At room temperature, the accumulated amount of sericin released from the silkworm cocoon reaches 8 to 12 μg/ml over a period of 38 days^14^. In this study, the concentration of released sericin was 3 to 10 μg/ml over a period of 4 h (Fig. 2A). The difference in the released amount might be due to differences in the extraction method. The molecular weight of sericin and the efficiency of extraction changed according to the extraction methods^30^. Sericin has many beneficial effects, and sericin products are used in hair care, cosmetics, and biomaterials^31^. Soluble sericin is viewed as a biologically inert protein^32^. Because soluble sericin is released from the silkworm cocoon, the cocoon itself has been used in biomedical applications^11,33^. Therefore, the silkworm cocoon should be considered to be a drug carrier that releases sericin slowly at body temperature.

The released protein from each silk mat was identified as sericin (Supplementary Fig. 1). When sericin was administered to RAW264.7 cells, the expression level of TNF-α increased in a dose-dependent manner (Fig. 3A). Sericin can also increase the levels of inflammation-related cytokines^34^. Because the concentration of released protein was highest in the layer 1 group, the expression level of TNF-α in RAW264.7 cells was also highest in the layer 1 group (Fig. 3B). Because the concentration of released protein was low in the layer 2 and the layer 3 groups, the expression level of TNF-α in RAW264.7 cells was also low in the layer 2 and the layer 3 groups (Fig. 3B). In this study, a low level of TNF-α could increase ALP and Runx2 gene expression (Fig. 4). Runx2 is an important transcription factor in osteogenesis and can increase the expression level of genes associated with bone formation, such as BMP and Wnt, among others^35^. When a low level of TNF-α (10 ng/mL) was administered to dental pulp-derived stem cells, osteogenic differentiation was induced via the nuclear factor-κB (NF-κB) signalling pathway^36^. However, a higher concentration of TNF-α (100 ng/mL) reduces the expression of BMP-2, ALP and Runx2^37^. Alginate dressing can accelerate wound healing by elevating the level of TNF-α^38^. However, this trend was not evident for 10 ng/mL of TNF-α administration (Fig. 4), perhaps due to the difference in the cell type used. Considering that the induced concentration of TNF-α was dependent on the administered concentration of sericin, new bone formation from each silk mat was expected to be different according to the layer of the silkworm cocoon. Indeed, a low concentration of sericin (<0.0001%) increased ALP activity and Alizarin red staining in MC3T3-E1 cells, but a high concentration of sericin (>0.001%) strongly inhibited ALP activity and Alizarin red staining^14^.

**Figure 7:**
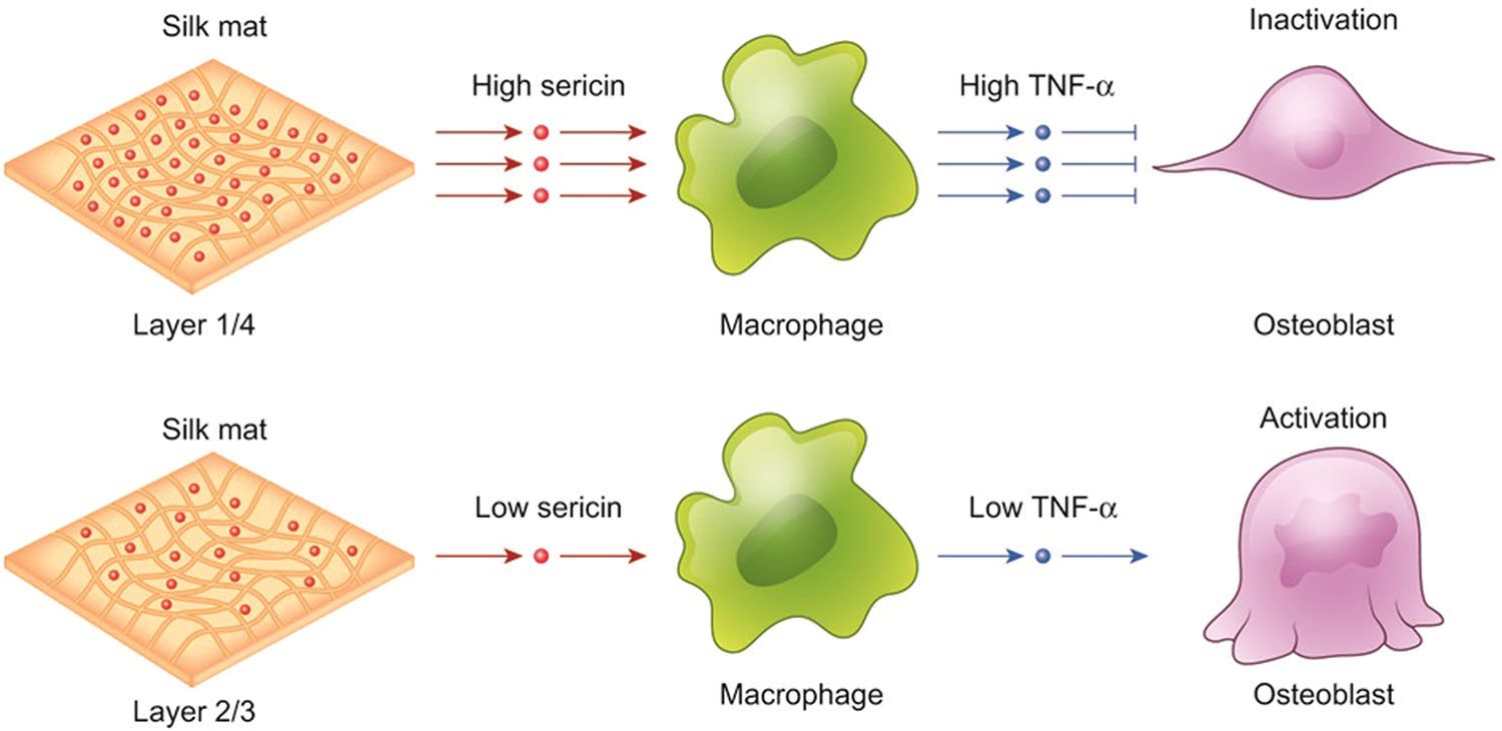
Schematic drawings of the proposed mechanism. Layer 1/4 had higher sericin than layer 2/3. Consequently, released sericin fragments are expected to be higher in layer 1/4 than in layer 2/3. The behaviour of macrophage with sericin differed according to the concentration. A high concentration of sericin increased the level of TNF-α expression in macrophages compared with a low concentration of sericin. The expression of osteogenic genes in osteoblasts was dependent on the concentration of applied TNF-α.

In this study, the bone volume at 8 weeks after surgery was highest in the layer 2/3 group (Fig. 5). When a high concentration of TNF-α (5 μg) was loaded into the layer 2/3 group, the gain in bone volume was decreased (Fig. 5). TNF-α is involved in inflammation and the development of osteoclasts^39^. Accordingly, TNF-α is associated with foreign body-induced inflammation and bone loss^40^. Prosthetic hip or knee joint-related osteolysis is induced by wear debris from the prosthetics^41^. Unlike metallic wear debris, sericin released from silk mat is a degradable protein. However, the released concentrations of sericin differed among silk mats, and this difference seemed to influence bone regeneration via the TNF-α-mediated pathway (Fig. 7). TNF-α alone inhibits the differentiation of tendon-derived stem cells, but TNF-α increases the proliferation of stem cells in combination with TGF-β1^42^. Osteogenic differentiation of dental pulp stem cells can be increased by the application of TNF-α via the NF-κB pathway^43^. In the initial inflammatory phase, TNF-α works together with BMPs to induce bone regeneration^44^. Selected inflammatory pathologic conditions, such as ankylosing spondylitis^45^ or periostitis ossificans^46^, can result in excessive bone formation. Induction of controlled inflammation is a strategy for the development of bone graft materials^47,48^, and a short duration of inflammation can increase new bone formation^49^. Concurrent treatment of TNF-α combined with BMP-2 results in higher bone formation than treatment with a single cytokine^44^. If TNF-α is administered locally at 24 h after injury, fracture healing is accelerated^50^. Macrophages can be used during the production of TNF-α because macrophages are important in alloplast-induced bone formation^51^. A high concentration of TNF-α (100 ng/ml) decreased alkaline phosphatase activity and reduced the expression of osteogenic genes in bone marrow-derived cells^18^. Runx2 is regulated by the WNT pathway and is an important transcription factor for bone formation^52^. Administration of a high concentration of TNF-α (10 ng/ml or 100 ng/ml) increases gene expression of BMP-2, but suppresses osteogenic differentiation in bone marrow cells^53^.

Drug delivery platform for tissue engineering is important for therapeutic effects and reduced complication associated drug administration^54^. Classic materials for tissue engineering has many limitations and natural biomolecules have been illuminated as biocompatible and bio-degradable platform^55^. Fragmented sericin (15–60 kDa) does not induce the antibody formation in the rabbits and the controlled release of sericin accelerate tissue regeneration^56^. Smart materials can be defined as “being responsive or instructive, or a combination of both these qualities”^57^. Smart materials let cells to do as a desired manner. These smart materials can overcome the limitations of conventional materials and silk based natural polymers are example of smart materials^58^. Because silk mat is produced by an eco-friendly method, it has been considered for use in clinical applications, but no guidelines exist for its use in clinical applications. In this study, we demonstrated that TNF-α induction in macrophages was dependent on the concentration of sericin administered and could influence bone regeneration. Accordingly, silk mat from silk cocoon could be considered fragmented sericin secreting carrier and the level of sericin secretion was associated with TNF-α induction and bone regeneration.

## Materials and Methods

This study was approved by Gangneung-Wonju National University. All experiments were performed in accordance with the relevant guidelines and regulations.

### Preparation of the silk cocoon and scanning electron microscopic (SEM) examination

Silkworm cocoons originated from mulberry silkworms (*Bombyx mori*) supplied by the Rural Development Administration (Wanju, Korea). The cocoon was sliced, and four layers were separated with even thickness. The material was cut into 10 × 10 mm squares using micro-scissors. The separated layers were classified by their deposition of silk fibres on the cocoons. The innermost layer was set as layer 1, and the outermost layer was set as layer 4. The thickness of each layer was approximately 0.2 mm. The morphology of the separated layers was observed by SEM. Images were captured by the SEM (Hitachi, SU-70, Japan) at 5 keV.

### Attenuated total reflectance Fourier transform infrared (ATR-FT-IR) spectroscopy and physical strength test

ATR-FT-IR measurements were performed by the Korea Basic Science Institute (Daegu, Korea). Subsequent procedures were conducted in accordance with our previous publication^14^. The physical strength of each silk mat was tested by the Korea Institute of Industrial Technology (Gangneung, Korea). Samples were prepared with dimensions of 30 × 10 mm, and both sides were fixed for measurement.

### Protein release analysis and identification

The weight of each layer was 2 g. The layers were placed in 50 mL of saline at 25 °C and sonicated for 4 h. Supernatant was collected for the analysis. The protein concentration in saline was measured by spectroscopy at 280 nm. The control was bovine albumin.

The solution of released proteins was collected and applied to SDS-PAGE to examine the protein profile. After SDS-PAGE, all solutions were mixed with the same volume and subjected to two-dimensional gel electrophoresis. Subsequent experiments were referred to ProteomeTech Inc. (Seoul, Korea). Amino acid sequencing was performed for the protein spots identified in two-dimensional gel electrophoresis. After fragmentation with trypsin, LC MS/MS was used in protein identification.

### Cell culture

*In vitro* cell culture was conducted using C2C12 pre-osteoblast cells (ATCC, Manassas, VA, USA) and RAW264.7 cells (KCLB No. 40071). The cells were grown to 80% confluence in alpha-MEM (HyClone, Logan, Utah, USA) and Dulbecco’s modified Eagle’s medium-high glucose (PAA Laboratories, Linz, Austria), respectively, containing 1% penicillin/streptomycin (100X) supplemented with 10% autologous serum. The cells were maintained at 37 °C in an atmosphere of 5% CO_2_ and 99% relative humidity. Various concentrations of silk sericin or sericin released from the silkworm cocoon were applied to cultured RAW264.7 cells to evaluate the level of TNF-α expression. The effect of TNF-α on the expression level of osteogenic genes was tested in C2C12 cells. The number of cells seeded per well was 5 × 10^4^.

### Reverse transcription-polymerase chain reaction (RT-PCR)

Total RNA was isolated from C2C12 cells using the easy-BLUE™ (iNtRON Biotechnology, Kyungki-Do, Korea) RNA extraction reagent. cDNA was synthesized from total RNA using AccuPower RT PreMix (Bioneer, Daejeon, Korea) and was subsequently used in quantitative real time-PCR amplification using AccuPower®2X GreenStar™ qPCR MasterMix (Bioneer). The forward and reverse primers for the real-time PCR of mouse genes are listed as follows (5′ → 3′): GAPDH F: TCA ATG ACA ACT TTG TCA AGC and R: CCA GGG TTT CTT ACT CCT TGG, ALP F: CCA ACT CTT TTG TGC CAG and R: GGC TAC ATT GGT GTT GAG CTT TT, Runx2 F: TTC TCC AAC CCA CGA ATG CAC and R: CAG GTA CGT GTG GTA GTG AGT, Osteocalcin F: CTG ACA AAG CCT TCA TGT and R: GCG CCG GAG TCT GTT CAC, Osterix F: CCC ACC CTT CCC TCA CTC and R: CCT TGT ACC ACG AGC CAT. GAPDH was used as the normalization reference of each sample for quantification.

### Western blot and ELISA

To assess the inflammatory effect of the sericin released from each silk mat on RAW264.7 cells, 2.0 g of each silk net was dipped in 50 mL of normal saline and sonicated at 37 °C for 4 h, and the supernatant was subsequently collected. Prior to cellular treatment, high performance liquid chromatography (Agilent 1100 series, Agilent Technologies, Waldbronn, Germany) was performed on each leeched solution. The column was a non-coated and non-adherent silicate column, and the solvent was distilled water. The same volume of leeched solution from each net (750 μL) was applied to cultured RAW264.7 cells. Protein was collected at 2, 8 and 24 hours after administration. The collected proteins were mixed with reducing buffer, and the subsequent procedure was conducted as reported previously^54^. The antibody used in western blot analysis was purchased from Santa Cruz Biotechnology (cat#: sc-12744, Santa Cruz, CA, USA).

The cells were washed with PBS and scraped into PRO-PREP™ Protein Extraction Solution (iNtRON Biotechnology). Equal amounts of proteins were subjected to SDS-PAGE and subsequently electro-transferred onto a PVDF membrane. The membrane was blocked with 5% non-fat dry milk in Tris-buffered saline containing 0.1% Tween 20 and incubated with the relevant primary antibody, followed by incubation with the corresponding HRP-conjugated secondary antibody. Anti-osteocalcin antibody was purchased from Santa Cruz Biotechnology, and anti-Runx2 antibody was sourced from Medical & Biological Laboratories (Woburn, MA, USA). Anti-β-actin antibody and HRP-conjugated secondary antibodies were obtained from Santa Cruz Biotechnology. Labelled protein bands were visualized using the ECL reagent (Bio-Rad, Hercules, CA, USA), and luminescence was detected with Chemi-doc^®^ (Bio-Rad).

ELISA for TNF-α was performed for RAW264.7 cells. Silk sericin was applied to RAW264.7 cells at concentrations of 1, 5, and 10 ng/ml, and the supernatant was collected. ELISA was conducted using a commercially available kit (Cat#: KMC3011, Invitrogen, Carlsbad, CA, USA) and the detailed protocol supplied by the manufacturer.

### Animal experiments

This experiment was approved by the Institutional Animal Care and Use Committee of Gangneung-Wonju National University, Gangneung, Korea (GWNU-2013-10). Sprague-Dawley rats at 12 weeks old (n = 25) were used in this experiment. Anaesthesia consisted of intramuscular injection with a combination of 0.5 mL Tiletamine and Zolazepam (125 mg/mL of 1:1 mixture; Zoletil; Bayer Korea, Seoul, Korea) and 0.5 mL xylazine hydrochloride (10 mg/kg body weight; Rompun; Bayer Korea, Seoul, Korea). After treatment with chemical disinfectant, the cranium area was shaved. An incision was made on the mid-sagittal area of cranium, and the parietal bone was exposed by sharp subperiosteal dissection. A dental-trephine burr with an 8 mm diameter was used in the preparation of a full thickness bony defect on the midline. Twenty-five animals were used in the comparative study of each layer of the silk mats (10 × 10 mm). The silk mat was placed on the calvarial defect. Because layer 2 and layer 3 had shown similar behaviour in the cellular experiments, layer 2 and layer 3 were mixed and grouped as layer 2/3. The control group did not receive any silk mat. To assess the effect of TNF-α on bone regeneration, 5 μg of recombinant rat TNF-α (#400-14, PeproTech) was loaded onto each silk mat from layer 2/3 and set as the layer 2/3 + TNF-α group. For the four types of silk mats (i.e., layer 1, layer 2/3, layer 4 and layer 2/3 + TNF-α), each type of mat was placed on the bone defect. Each rat was individually caged and fed, and after 8 weeks, all animals were sacrificed.

### Micro-computerized tomography and histomorphometric evaluation

The calvarial specimens (12 × 12 × 3 mm) were sent to the Korea Basic Science Institute (Ochang, Korea) for micro-CT analysis. The subsequent procedure was performed in accordance with the description in our previous publication^28^. In brief, an animal PET/CT/SPECT system (Inveon Siemens, Malvern, PA, USA) was used in analysis. The scanned images were reconstructed using the supplied software (Inveon Siemens). The size of the initial surgical defect was referenced to determine the region of interest (ROI). The bone volume (BV) in the ROI was calculated by setting a threshold level of 25% bone standard.

After image analysis by micro-CT, the specimens were processed for histological analysis by decalcification. The specimens were cut into two pieces on the mid-sagittal suture and were embedded to show the sagittal section. Collagen staining was performed with a commercially available kit (ab150681, Abcam, Cambridge, UK). The subsequent procedure was applied in accordance with the manufacturer’s protocol. Deparaffinized sections were placed into picro-sirius red solution for 1 h and washed twice in acetic acid solution. To determine the level of TNF-α expression in the tissue, immunohistochemical staining was performed using anti-TNF-α antibodies (Santa Cruz) at a dilution ratio of 1:50^59^.

### Statistical analysis

The analysis of variance test was used to compare multiple groups, and Bonferroni’s method was selected for the post hoc test. The significance level was P < 0.05.

### Data availability statement

Data sharing not applicable to this article as no datasets were generated or analysed during the current study.

### Ethical approval and informed consent

This study was approved by Gangneung-Wonju National University. Animal experiment was approved by the Institutional Animal Care and Use Committee of Gangneung-Wonju National University, Gangneung, Korea (GWNU-2013-10). All experiments were performed in accordance with the relevant guidelines and regulations.

## Electronic supplementary material

Supplementary data
