# Supplementary material for: Bone regeneration is associated with the concentration of tumour necrosis factor-α induced by sericin released from a silk mat
Source: Sci Rep. 2017 Nov 14;7:15589. doi: 10.1038/s41598-017-15687-w (PMC5686134; doi:10.1038/s41598-017-15687-w)
Supplement: Supplementary file 1 — Supplementary data [file 41598_2017_15687_MOESM1_ESM.doc]

**Bone regeneration is associated with the concentration of tumour necrosis factor-α induced by sericin released from a silk mat**

You-Young Jo^1^, HaeYong Kweon^1^, Dae-Won Kim^2^, Kyung-Hwa Baek^3^, Min-Keun Kim^4^, Seong-Gon Kim^4, *^, Weon-Sik Chae^5^, Je-Yong Choi^6^,*, and Horatiu Rotaru^7^

^1^Sericultural and Apicultural Division, National Institute of Agricultural Science, RDA, Wanju 55365, Republic of Korea

^2^Dept. of Oral Biochemistry, College of Dentistry, Gangneung-Wonju National University, Gangneung 28644, Republic of Korea

^3^Dept. of Oral Pharmacology, College of Dentistry, Gangneung-Wonju National University, Gangneung 28644, Republic of Korea

^4^Dept. of Oral and Maxillofacial Surgery, College of Dentistry, Gangneung-Wonju National University, Gangneung 28644, Republic of Korea

^5^Analysis Research Division, Daegu Center, Korea Basic Science Institute, Daegu 41566, Republic of Korea

^6^School of Biochemistry and Cell Biology, BK21 Plus KNU Biomedical Convergence Program, Skeletal Diseases Analysis Center, Korea Mouse Phenotyping Center (KMPC), Kyungpook National University, Daegu 41944, Korea

^7^Department of Cranio-Maxillofacial Surgery, “Iuliu Hatieganu” University of Medicine and Pharmacy, Cluj-Napoca 400001, Romania

*Corresponding author

E-mail address: kimsg@gwnu.ac.kr, Fax: +82-33-641-2477 and jechoi@knu.ac.kr

**Supplementary Figure 1. Identification of soluble proteins in silk mat**

**
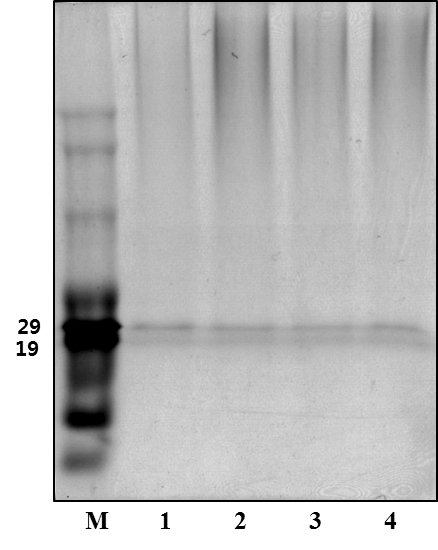
**

(A) The result of SDS page demonstrated that two protein bands were identified and their size was approximately 27 kDa and 19 kDa. There was no prominent difference in the profile of protein bands among silk mat layers. There were dragged proteins above 29 kDa area.

(B) The released proteins from silk mat were underwent two-dimensional electrophoresis. In two-dimensional electrophoresis, there was no prominent protein spot. Large protein clusters were identified in pH 3 and their molecular weight was between 15 and 50 kDa. Selected area of proteins (red rectangle) were treated by trypsin and underwent LC MS/MS. They were fragmented sericin. Other proteins were not identified.

**Supplementary Figure 2. Western blot assay for Runx2 and osteocalcin (OCN)**


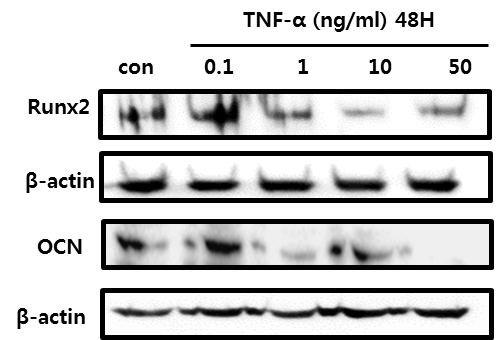


Western blot assay showed increases in the protein expression of Runx2 and OCN in C2C12 cells incubated with low concentrations of TNF-α (0.1 and 1 ng/ml) at 48 h.

**Supplementary Figure 3. Full length blot of Figure 3**


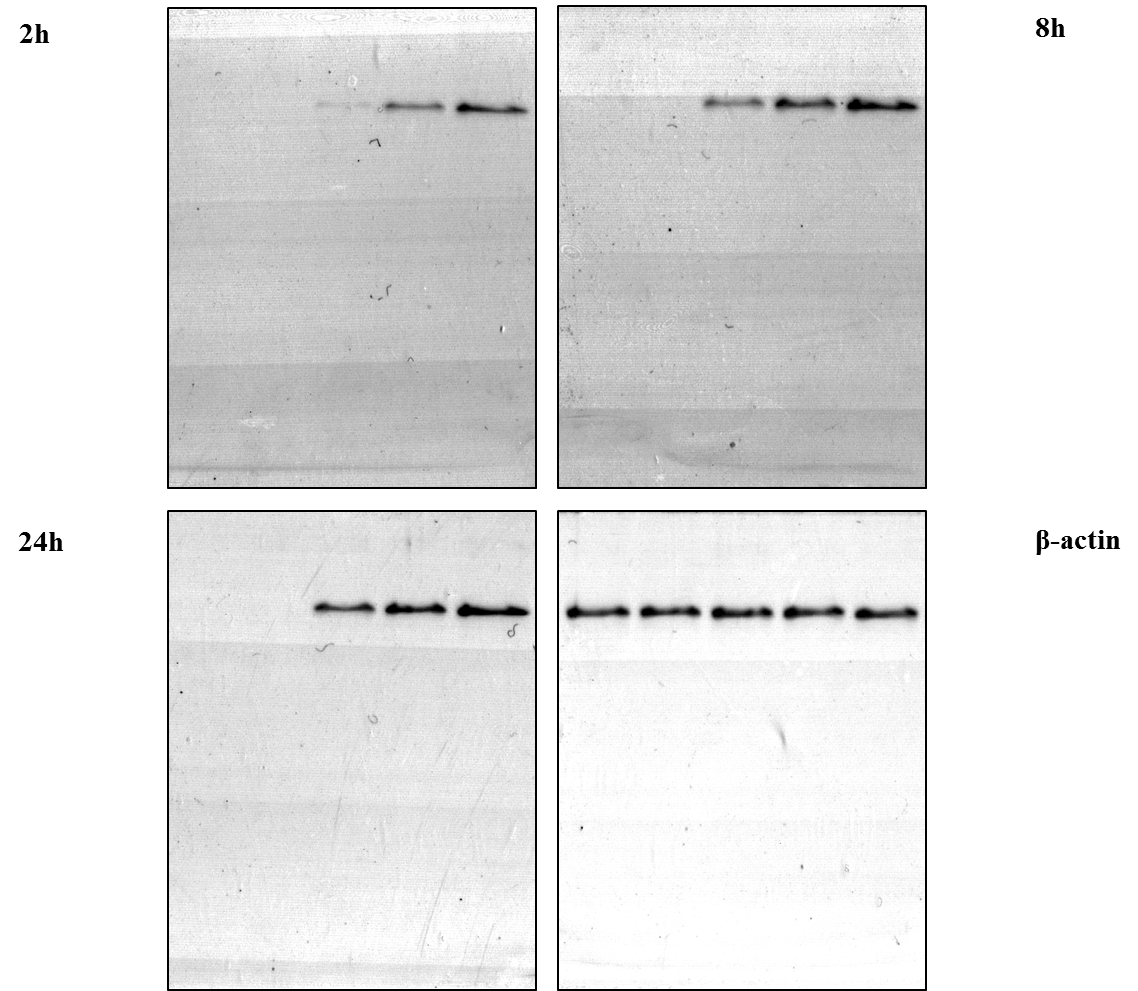


(A) The expression level of TNF-α was increased by the administration of sericin in a dose-dependent manner. There were 5 lanes in each blot and they were untreated control, 1, 3, 5, and 7 ng/ml of sericin treatment from left.


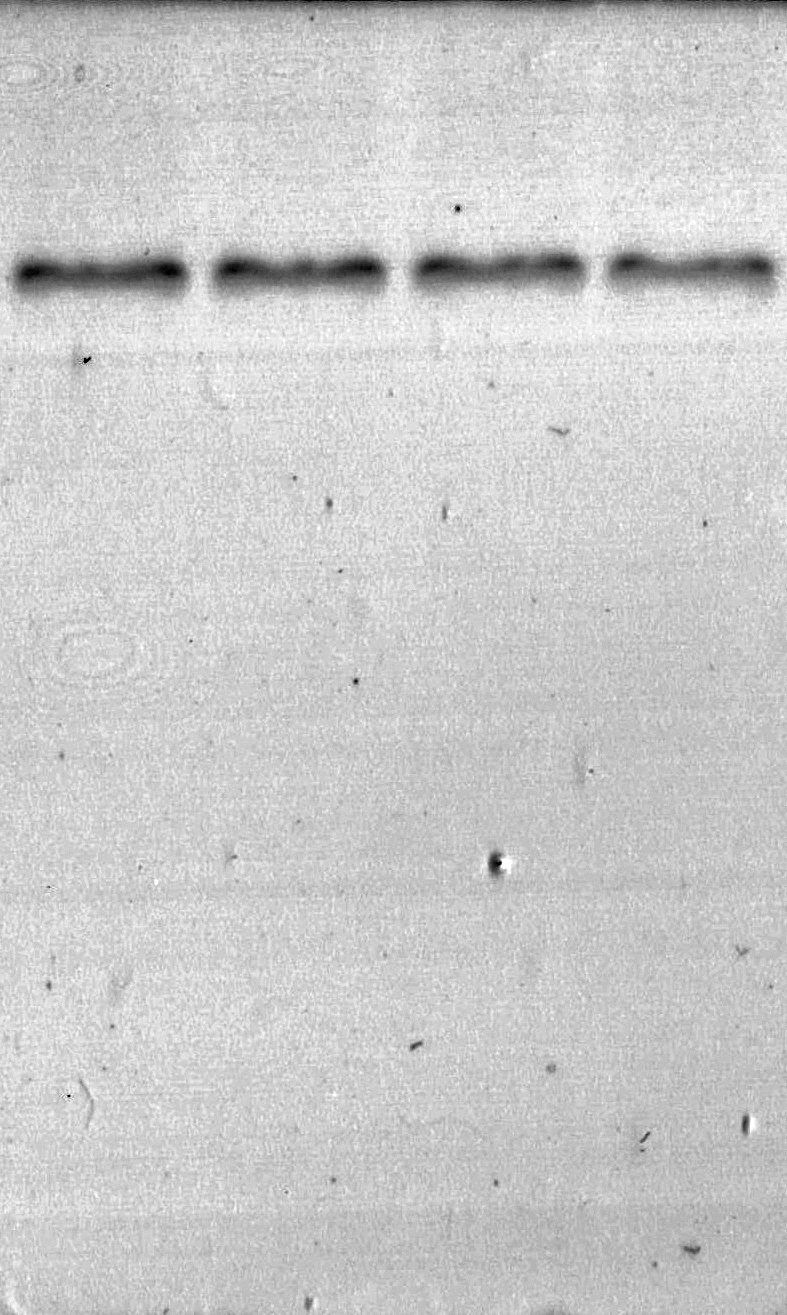


**2hour**

**8hour**

**24hour**

**β-actin**


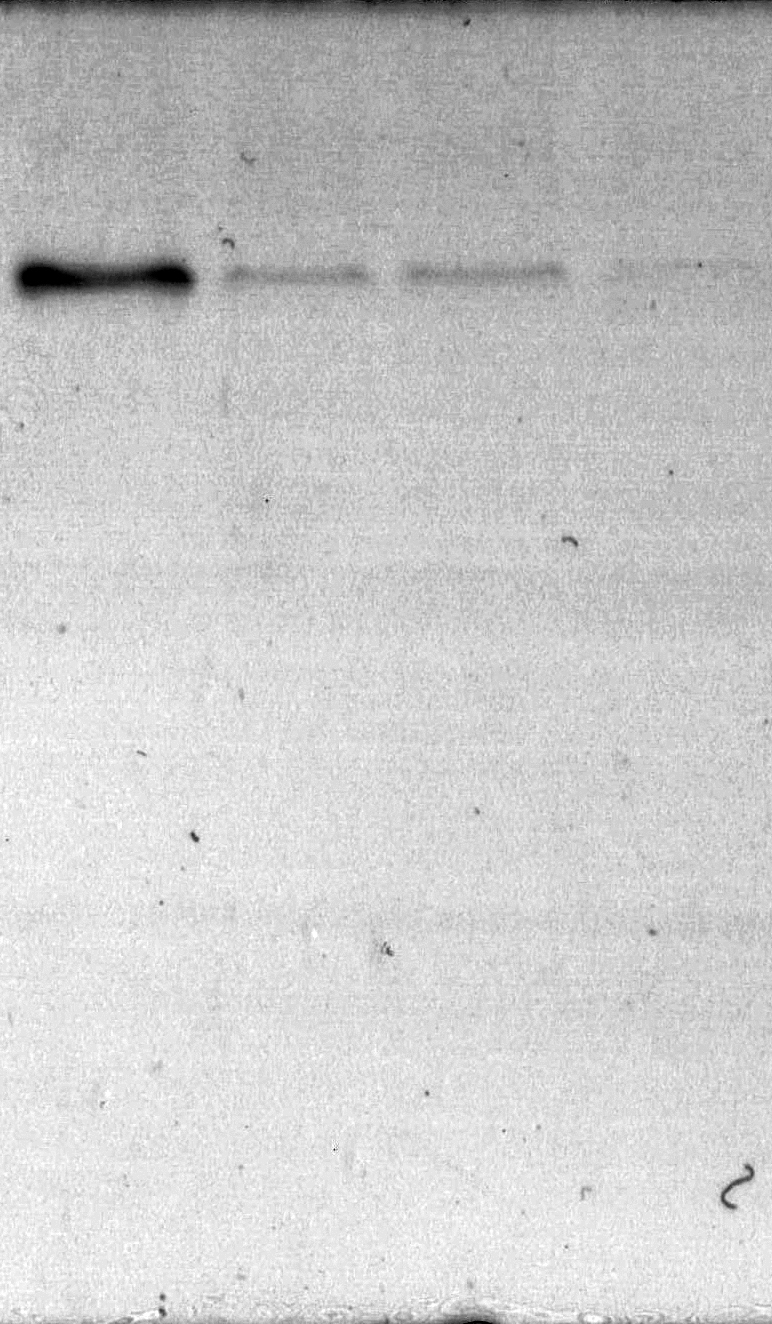

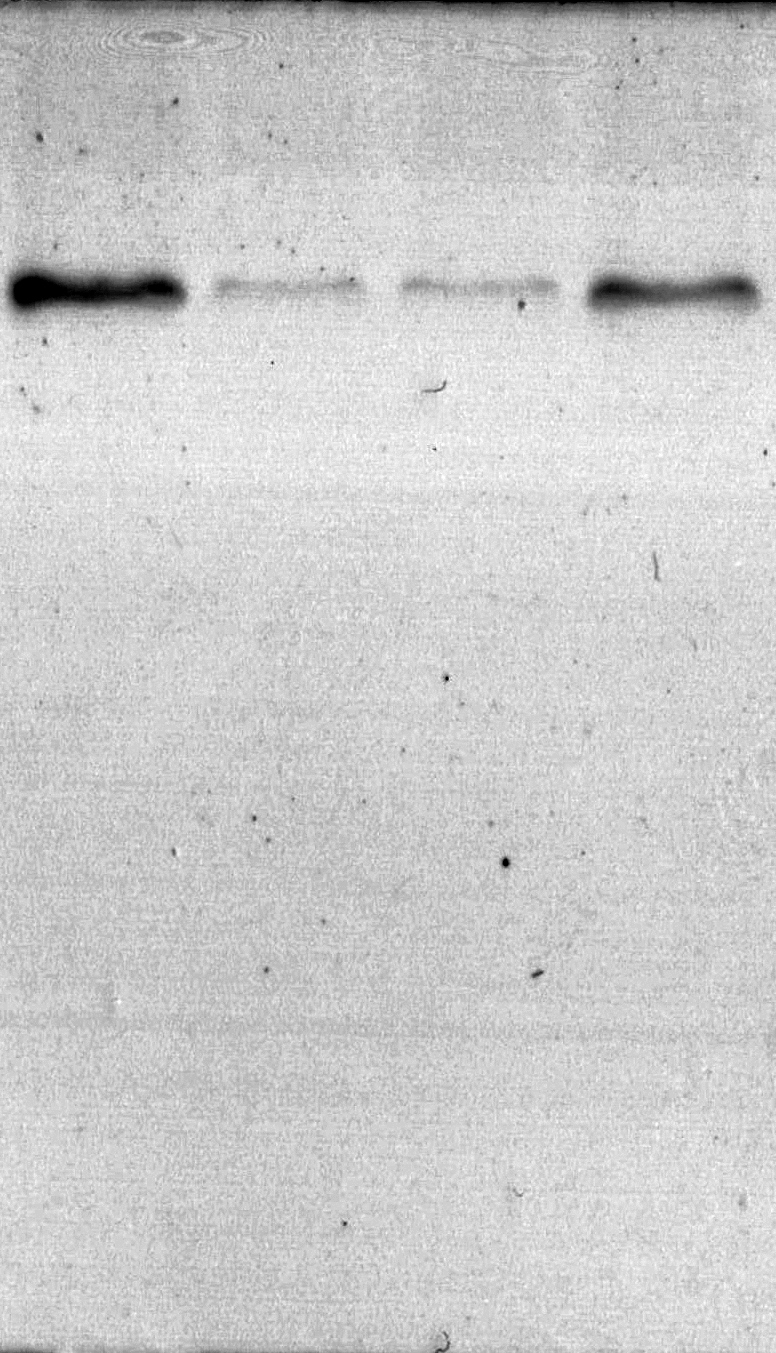

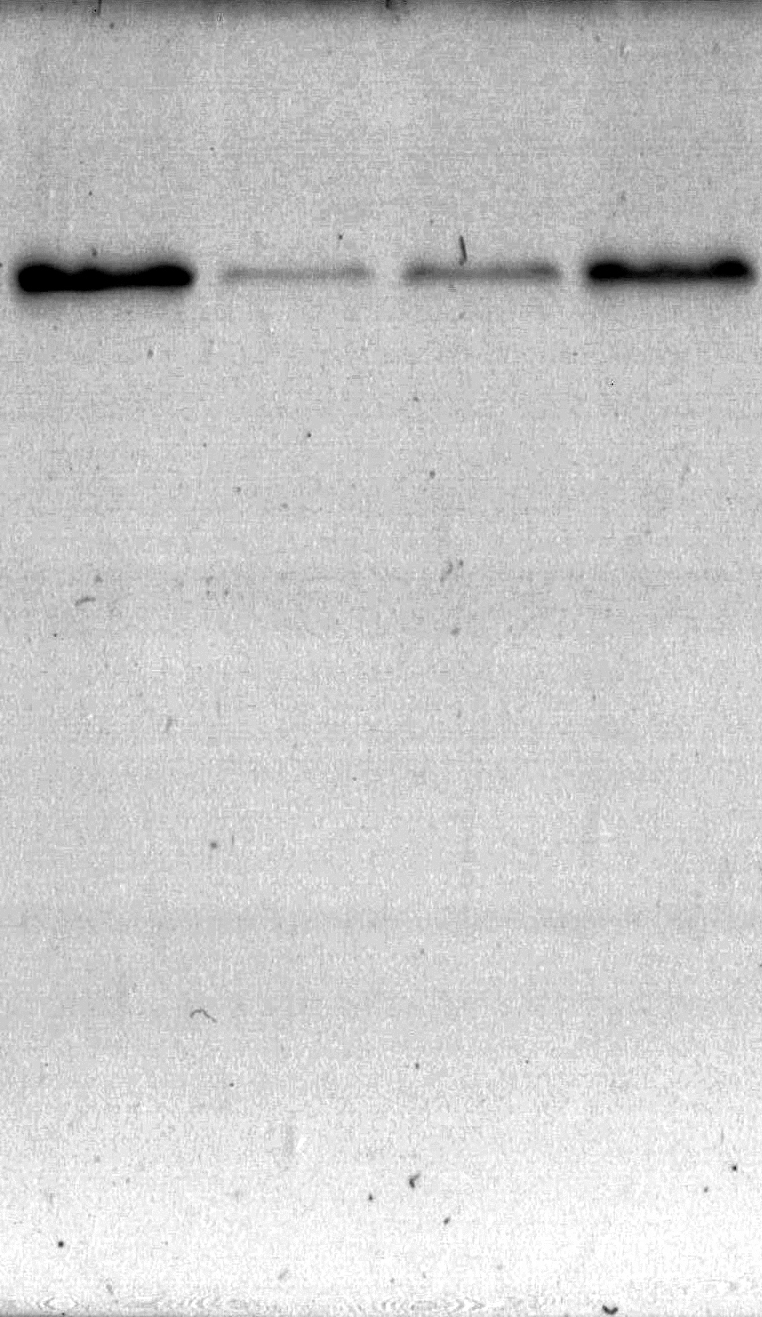


(B) The protein solution of silk mat from each layer was administered to RAW264.7 cells. There were 4 lanes in each blot and they were layer 1, 2, 3, and 4 group from left. Because the concentration of dissolved protein was different for each silk mat, the expression level of TNF-α in each silk mat was also different. The expression level of TNF-α was highest in the layer 1 group followed by that of the layer 4 group.
